# Patients With Reflux Esophagitis Possess a Possible Different Oral Microbiota Compared With Healthy Controls

**DOI:** 10.3389/fphar.2020.01000

**Published:** 2020-07-07

**Authors:** Baoyong Wang, Yu Zhang, Qiaofei Zhao, Yifan Yan, Tian Yang, Yanli Xia, Hongwei Chen

**Affiliations:** Department of Gastroenterology, Luoyang Central Hospital Affiliated to Zhengzhou University, Luoyang, China

**Keywords:** reflux esophagitis, oral microbiota, dysbiosis, high-throughput sequencing, biomarker

## Abstract

**Background and Aim:**

Reflux Esophagitis (RE) is caused by a variety of factors including anatomical and functional alterations involved in the pathogenesis. Oral microbiota is influenced by many factors such as heredity, nutrition, environments and host conditions, but little is known about relationship between oral microbiota and RE. The aim of this study was to explore whether the oral microbiota is changed in patients with RE.

**Methods:**

To clarify this correlation, fresh saliva samples from all subjects were collected and then oral microorganism diversity was analysed in 55 patients with RE and 51 controls *via* hypervariable tag sequencing and analyzing the V3–V4 region of the 16S rDNA gene.

**Results:**

There was no difference found in oral microbial diversity between RE patients and healthy controls by Shannon diversity index (p=0.60) and Simpson diversity index (p= 0.38). The abundance of *Proteobacteria* was lower, but *Bacteroidetes* was higher in patients with RE at the phylum level. At the genus level the abundances of *Prevotella*, *Veillonella, Megasphaera*, *Peptostreptococcus*, *Atopobium*, *Oribacterium*, *Eubacterium*, and *Lachnoanaerobaculum* were increased, while *Neisseria, Streptococcus, Rothia, Granulicatella, Gemella, Aggregatibacter, Treponema, Campylobacter, Filifactor, Corynebacterium*, and *Lactivibrio* were decreased in RE patients than the controls.

**Conclusions:**

Our study suggested oral microbial dysbiosis in patients with RE, and identified bacterial species with potential biomarker significance. Further studies are required to understand role of oral microbial dysbiosis in the pathogenesis of RE.

## Introduction

As one of the most common outpatient diseases, gastroesophageal reflux disease (GERD) has attracted increased attention due to its growing burden. Recent studies have reported that the prevalence of GERD in North America ranges from 18% to 28% (El-Serag et al., 2014; Peery et al., 2015). As a subtype of GERD, reflux esophagitis (RE) is usually caused by the gastric and duodenal contents refluxing into the esophagus and oral cavity, with injured lower esophageal mucosa including erosion and ulcer observed by electronic endoscope (Vakil et al., 2007; Katz et al., 2013; Chuang et al., 2017).

By tradition medicine samples of stools of volunteers are subject to analysis of the gastrointestinal microbiota. However, oral bacterial species are reported in gut microbial samples (Franzosa et al., 2014; Schmidt et al., 2019). Furthermore, the salivary microbiota changed in age and many diseases, such as oral diseases, tumor, systemic lupus erythematosus, type 2 diabetes, obesity and so on (Chen and Jiang, 2014; Burcham et al., 2020; Li et al., 2020). Saliva is no doubt an simple and noninvasive alternative to such sampling strategies in clinical work (Raj et al., 2018). Therefore, saliva would be a feasible alternative to local samples in future researches of the microbiota in oral and disease (Belibasakis et al., 2019).

More recently, Rubenstein JH et al. characterized that reflux symptoms of GERD increased the risk of esophageal adenocarcinoma about fivefold (Rubenstein and Taylor, 2010). Because of reflux the difference in esophageal microecology has been reported, which may associate with persistent and progressive esophageal diseases. Recently there was one report on changes at the genera level, suggesting the dysbiosis Prevotella, Helicobacter, and Moraxella in the distal esophageal RE patients (Yu et al., 2019). Both esophagus and oral cavity is injured during the progression of reflux, so the oral microbiota may be changed in RE patients. Moreover, the oral microbiota influences the esophageal microbiome (Zh et al., 2004), due to migration of oral bacteria to the esophagus (Lawson and Coyle, 2010), and then may lead to esophageal diseases including RE. But, it is yet unknown whether oral microbiota may be changed in patients with RE.

We hypothesized that oral microbiota influence development of RE. We conducted a prospective study to analyze the composition of oral microflora in the RE patients compared with healthy controls using high-through put DNA sequencing.

### Patients

A total of 55 RE patients and 51 age-and sex-matched healthy volunteers (controls) were enrolled prospectively from July to December 2018 in the study. All the RE patients with typical symptoms, such as heartburn and regurgitation were in accordance with the endoscopic criteria of Los Angeles Grade and taken no drugs as treatment from Luoyang Central Hospital affiliated to Zhengzhou University (Henan Province, China). Several studies have shown that proton pump inhibitors(PPIs) can alter the micro ecological structure of the gastroesophageal tract (Sanduleanu et al., 2001; Amir et al., 2014; Gall et al., 2015). Healthy controls met criteria for a normal esophagus under endoscopy and no symptoms of heartburn and regurgitation, which excluded RE in particular. Only those volunteers who had not used glucocorticoids, antibiotics, PPIs and immunosuppressive drugs within 6 months were enrolled. The exclusion criteria were the presence of other serious diseases, such as malignancy, immunodeficiency or any other immunological disorder, metabolic diseases, any other serious internal disease. Pregnant, nursing women and people with a gastrointestinal tract surgery history were also excluded. This study was approved by the ethics committees of the Luoyang central hospital affiliated to Zhengzhou University.

### Questionnaire Investigation

A food frequency questionnaire was used to investigate subjects’ daily diet intake during the previous year. Additionally, information, such as clinical characteristics and living habits and demographic characteristics were collected. Clinical data of volunteers, including gender, Body mass index (BMI), and age were recorded before sample acquisition. All subjects were also questioned about smoking, alcohol drinking, fibre intake, salt intake, sugar intake, fat intake, meat intake, and vegetarian preference over the past 6 months. The frequency of consumption of cigarettes, alcohol was evaluated as follows: none (more than 1 month), occasionally (once a month and more than once a week), sometimes (once a week and more than 3 days), frequently (every 3 days or less than 3 days).

### Microbial Sampling and DNA Extraction

Tooth-brushing was instructed to refrain from the night before the sampling until the end of examination. Each person was not allowed from eating and drinking for at least 2 h prior to the examination. Fresh saliva samples from all subjects were collected in sterile graduated test-tubes after the volunteers rinsed their mouths thoroughly three times for 3 min with 10 ml scope mouthwash (Hayes et al., 2000; Calle et al., 2002) and then immediately frozen at –80°C within 30 min to keep highly stable over time (Costello et al., 2009; Zhou et al., 2013; Belstrøm et al., 2016). The samples were conveyed to Realbio Technology (Shanghai, China) for high-throughput sequencing. The V3–V4 hypervariable regions of the bacterial 16S rDNA gene sequences were amplified with primers F341F (5’-ACTCCTACGGGRSGCAGCAG-3’) and R806R (5’- GGACTACVVGGGTATCTAATC-3’) by PCR (GeneAmp 9700, Applied Biosystems, Foster City, CA, USA).

### High-Throughput Sequencing

High-fidelity amplification used by KAPA HiFi Hotstart ReadyMix PCR kit. NanoDrop 2000 spectrophotometer (Thermo Fisher Scientifc, Waltham, MA, USA) and 2% agarose gel electrophoresis assessed the quality of amplicons. The sequencings of 16S rDNA gene amplifcation products were detected by Illumina Hiseq 2500 instrument (Illumina, San Diego, CA, USA) with 2×250 base pair (bp) paired-end (PE) sequencing at Realbio Technology.

### Sequence-Based Microbial Analysis

Pandaseq was used to assemble overlapping end paired-end reads. The length of quality control retained sequence ranged from 220 to 500 nt, the average sequence score of > 20, and N bases read were < 3. The 16S rDNA sequences were divided into operational taxonomy units (OTUs) by the average neighbor algorithm. In order to calculate the downstream diversity measurements (alpha and beta diversity analysis), sequences with distance-based similarity of > 97% were allocated to the same OTU by USEARCH after removal of singletons. In addition, subsampling of 22,934 readings was extracted from each sample under condition of sequencing at a sufficient depth. RDP database and classifier (RDP, http://rdp.cme.msu.edu) were used to classify and distribute bacteria taxonomy. To detect the current sequencing depth, QIIME was used to generated Rarefaction curves (Caporaso et al., 2010). Shannon and Simpson diversity indices stood for species abundance in a single sample and were used to represent the α diversity and we compared between the two groups by Kruskal-Wallis rank sum test and Wilcoxon rank sum test using R3.1.0. Based on phylogenetic distance, the community was compared by the weighted UniFrac metric to reflect beta diversity (Lozupone et al., 2007). Using R3.1.0 multi-response permutation procedure (MRPP) analysis, Principal Co-ordinates analysis (PCoA) was used for evaluating distance matrix between each pair of samples. Furthermore, heatmap was also applied. The significance of sample clustering was tested by one-way analysis of similarities (ANOSIM) based on the UniFrac phylogenetic distance. The differences of abundant bacterial taxa between the two groups were performed by Linear discriminant *analysis* Effect Size (LEfSe) analysis. The taxa only reached a log linear discriminant analysis (LDA) score > 2, which were considered ultimately. If the Benjamini and Hochberg false discovery rate test (FDR) q value is > 0.1, FDR test was applied and the 95% confdence intervals (CI) wad calculated.

### Statistical Analysis

Clinical data and general date of patients was analyzed by SPSS 20.0 statistical software. Categorical variables were performed by Pearson’s Chi-square test and quantitative variables were performed by Student’s T-test. All *p* values of our study were bilateral, and a *p* less than 0.05 was considered to be statistically significant.

## Results

### The Basic Characteristics of RE and Healthy Groups

The demographic characteristics of RE group and healthy group are shown in **Table 1**. There was no significant difference in age, gender, body mass index (BMI, kg/m^2^), smoking, alcohol drinking, and fibre intake of participants. A total of 55 patients with RE and 51 controls were enrolled. No patient quitted and no data was excluded during the study. Moreover, all the saliva samples met the criterion of analysis in the study.

**Table 1:** Clinical characteristics of Reflux Esophagitis (RE) patients and healthy controls.

Characteristics	RE patients	Healthy controls	*p* value
Gender Female Male	30 25	24 27	0.441
Age,years, mean ± SD	52.55 ± 14.86	48.94 ± 12.01	0.175
Body mass index(BMI, kg/m^2^)	24.54 ± 3.23	25.57 ± 1.55	0.235
Smoking None or occasionally sometimes or frequently Alcohol drinking None or occasionally sometimes or frequently Fibre intake Low fibre intake High fibre intake Salt intake Low salt intake High salt intake Sugar intake Low sugar intake High sugar intake Fat intake Low fat intake High fat intake Meat intake Low meat intake High meat intake Vegetarian preference Low Vegetarian preference High Vegetarian preference	37 18 45 10 20 35 27 28 29 26 29 26 27 28 24 31	35 16 44 7 14 37 32 19 31 20 32 19 26 25 27 24	0.881 0.532 0.326 0.157 0.403 0.297 0.846 0.338

SD, standard deviation.

### Diversity of the Oral Microbiota in Patients With RE and Healthy Controls

**Figure 1:**
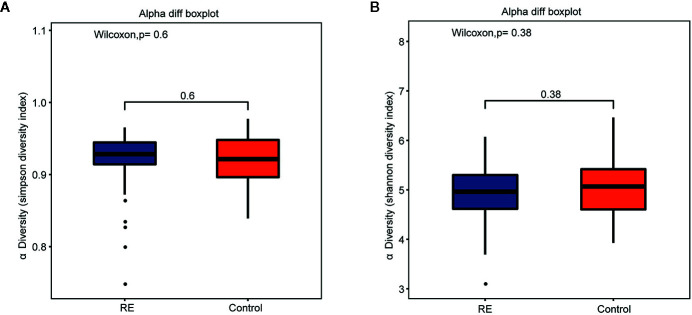
Comparisons of alpha diversity indices between Reflux Esophagitis (RE) and healthy controls. There was no great significance between RE and healthy controls in Simpson diversity index (**A**, *p*=0.60) and Shannon diversity index (**B**, *p*=0.38).

**Figure 2:**
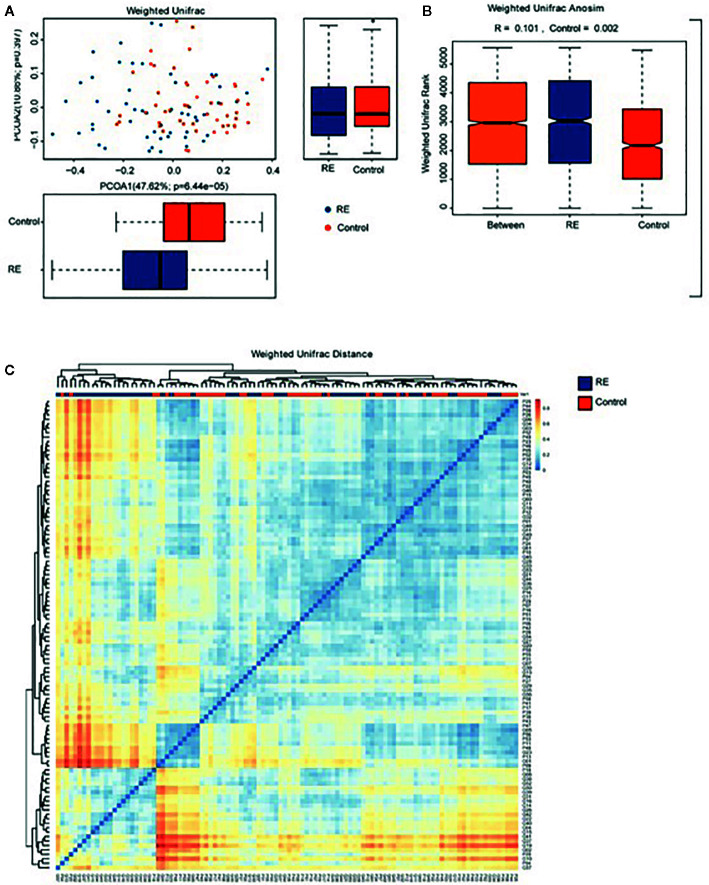
Comparison of the oral microbiome between Reflux Esophagitis (RE) and healthy controls. **(A)** Difference of relative abundances of operational taxonomy units (OTUs) between RE groups and the controls groups was evaluated by Principal coordinates analysis (PCoA) plot by using weighted UniFrac distance. **(B)** One-way analysis of similarities (ANOSIM) evaluated the difference of the two groups; **(C)** Heatmap showing difference from Weighted UniFrac phylogenetic distance matrices in the two groups.

Compared with controls, the RE patients displayed no significant change in microbial diversity as calculated by Simpson diversity index (*p*=0.60) and the Shannon diversity index (*p*= 0.38) (**Figures 1A, B** **)**. Principal coordinates analysis (PCoA) *via* weighted UniFrac distance matrix was used to assess contrast based on OTUs with different relative abundances. Our study showed that there were two tendencies including a tight cluster in controls (**Figure 2A**, red dots) and a disperse cluster in RE groups (**Figure 2A**, blue dots) in the PCoA plot. To study the oral microbes of subjects, we calculated UniFrac phylogenetic distances of the microbial composition among the patients and healthy volunteers. The significant difference of the two group was confirmed by one-way analysis of similarities (ANOSIM) (R=0.101, *p*=0.002) (**Figure 2B**). To approve a trend of difference between the two groups, heatmap was also used to show that the significant difference of each sample composition was obvious (**Figure 2C**).

### Discriminative Bacterial Taxa Between RE Patients and Healthy Controls

**Figure 3:**
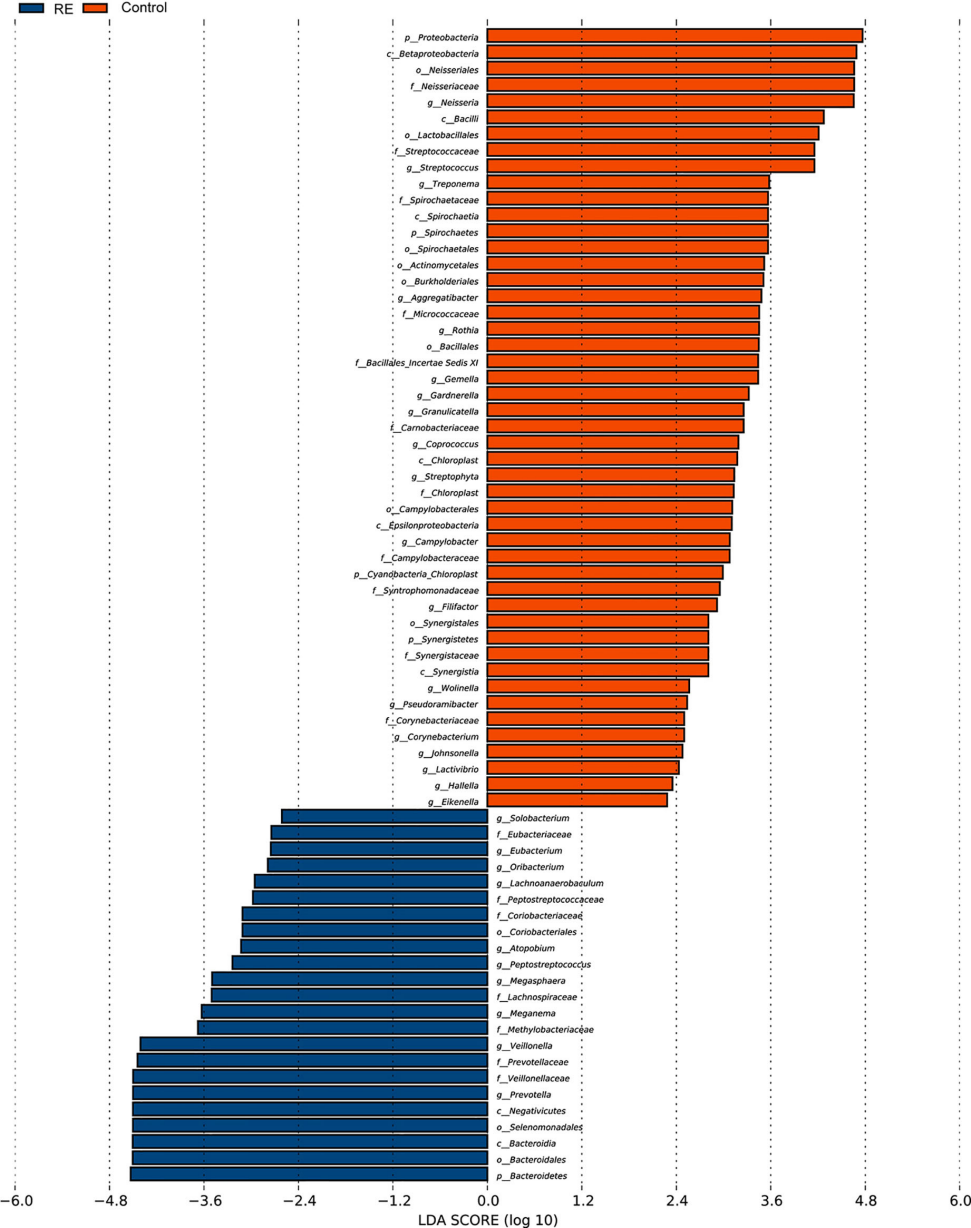
Bacterial taxa diversities between Reflux Esophagitis (RE) and healthy controls. Bacterial taxa were detected by LEfSe (*p*<0.05, linear discriminant analysis (LDA)>2 logs). Twenty-three texa were discovered to be enriched in patients with RE (*blue bars*) compared with controls, and 48 texa were discovered to be enriched in Controls (*red bars*) compared with patients with reflux esophagitis.

**Figure 4:**
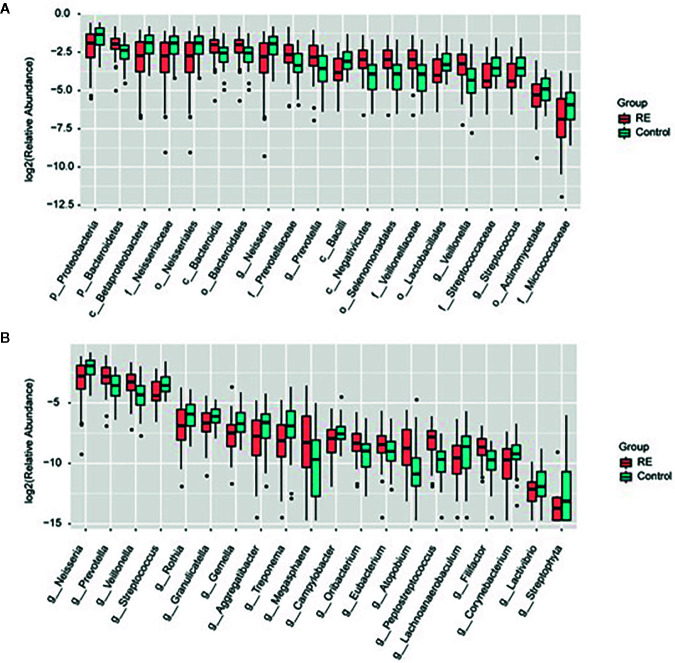
Relative abundance of the microbial taxa between Reflux Esophagitis (RE) and healthy controls. Relative abundance of top 20 diversely microbial taxa **(A)** and microbiota structure at the general level **(B)** in the RE groups and the controls groups were represented by box plot.

All oral saliva samples were analyzed by the LEfSe analysis and there were 1298 bacterial taxa to be analyzed, 667 of which bacterial taxa were the genus level. To explore the different taxa between RE patients and healthy controls, LEfSe analysis with LDA score >2 was performed. Our results suggested 71 taxa with significantly different abundancy. As shown in **Figure 3**, while compared to control group the RE patients displayed 48 decreased (red bars) and 23 increased taxa (blue bars). To investigate the differential abundance at the phylum, family, class, order, and genus levels we used Wilcoxon rank sum test and Kruskal-Wallis rank sum test, with results shown in **Table 2**. A standard of *p*-value <0.05, FDR q value < 0.1 was used to determine statistical significance. The 71 bacterial taxa above were identified to be different between RE patients and healthy controls, of which 29 taxa were at the genus level. The findings also showed the top 20 different oral microbes between RE patients and healthy controls (**Figure 4A**). The abundance of *Bacteroidetes* was higher, but *Proteobacteria* was lower in RE patients at the phylum level. We also found increased abundance of *Bacteroidia* and *Negativicutes* and decreased presence of *Betaproteobacteria* and *Bacilli* in RE patients at the class level. At the genera level the 20 different taxa in the oral saliva samples of the patients with RE were displayed in **Figure 4B**.The abundance of *Prevotella, Veillonella,Megasphaera, Peptostreptococcus, Atopobium, Oribacterium, Eubacterium*, and *Lachnoanaerobaculum* were higher and *Neisseria, Streptococcus, Rothia, Granulicatella, Gemella, Aggregatibacter, Treponema, Campylobacter, Filifactor, Corynebacterium*, and *Lactivibrio* were lower in RE patients than the controls at the genus level.

**Table 2:** Relative abundance of RE and healthy controls(Only *p*-values <0.01 are shown).

Taxon name	*p*-value	FDR q value
c__Bacilli	0.000	0.006
c__Bacteroidia	0.001	0.009
c__Betaproteobacteria	0.000	0.008
c__Chloroplast	0.012	0.086
c__Negativicutes	0.000	0.000
c__Spirochaetia	0.001	0.012
c__Synergistia	0.006	0.046
f__Bacillales_Incertae Sedis XI	0.000	0.004
f__Carnobacteriaceae	0.015	0.098
f__Chloroplast	0.012	0.086
f__Coriobacteriaceae	0.000	0.000
f__Corynebacteriaceae	0.0161	0.100
f__Lachnospiraceae	0.007	0.0520
f__Methylobacteriaceae	0.004	0.044
f__Micrococcaceae	0.011	0.082
f__Neisseriaceae	0.001	0.011
f__Prevotellaceae	0.002	0.023
f__Spirochaetaceae	0.001	0.012
f__Streptococcaceae	0.000	0.007
f__Synergistaceae	0.006	0.046
f__Syntrophomonadaceae	0.004	0.041
f__Veillonellaceae	0.000	0.000
g__Aggregatibacter	0.004	0.037
g__Atopobium	0.000	0.000
g__Corynebacterium	0.016	0.100
g__Eikenella	0.001	0.015
g__Gemella	0.000	0.004
g__Granulicatella	0.014	0.098
g__Hallella	0.001	0.009
g__Johnsonella	0.003	0.034
g__Lachnoanaerobaculum	0.000	0.000
g__Lactivibrio	0.001	0.013
g__Meganema	0.016	0.100
g__Megasphaera	0.001	0.012
g__Neisseria	0.001	0.011
g__Oribacterium	0.004	0.041
g__Peptostreptococcus	0.000	0.000
g__Prevotella	0.000	0.009
g__Pseudoramibacter	0.016	0.100
g__Rothia	0.010	0.082
g__Streptococcus	0.000	0.007
g__Streptophyta	0.012	0.086
g__Treponema	0.001	0.012
g__Veillonella	0.000	0.001
g__Wolinella	0.005	0.041
o__Bacillales	0.000	0.004
o__Bacteroidales	0.001	0.010
o__Burkholderiales	0.000	0.000
o__Campylobacterales	0.016	0.100
o__Coriobacteriales	0.000	0.000
o__Lactobacillales	0.000	0.009
o__Neisseriales	0.001	0.011
o__Selenomonadales	0.000	0.000
o__Spirochaetales	0.001	0.012
o__Synergistales	0.006	0.046
p__Bacteroidetes	0.000	0.004
p__Cyanobacteria/Chloroplast	0.012	0.086
p__Proteobacteria	0.001	0.010
p__Spirochaetes	0.001	0.012
p__Synergistetes	0.006	0.046

FDR, false discovery rate.

## Discussions

The balance in the structure of human micro-organisms plays an important role during pathogenesis and progress of diseases, and our research showed significant alterations in the oral microbes of RE patients as compared to healthy volunteers, although the alpha-diversity displayed no significant change.

Although many previous studies have suggested decreased diversity in the gut microbiota of patients with colorectal cancer (Wu et al., 2013), our study did not support a significant change in the diversity of oral microbiota in RE patients. Our data based on 55 RE patients and 51 healthy volunteers showed no significant change in microbial diversity as calculated by Simpson diversity index (*p*=0.60) and the Shannon diversity index (*p*= 0.38). A recent study on the microbial structure in distal esophagus cancer patients did not reveal a significant change in alpha-diversity, and another 16 s sequencing research on the esophageal microbiota of RE patients found no significant alteration on alpha-diversity (Sanduleanu et al., 2001). Thus, we propose that the change in diversity may be more dependent on the organ studied, although further investigation is required to confirm this.

Structure of microbiota in adjacent organs may be similar, according to previous studies. Using 16S rRNA gene sequencing, Grusell and colleagues found certain degree of similarity between the microbiota in the oral cavity, upper and lower esophagus (Norder Grusell et al., 2013). A previous research has suggested that distal esophageal microbiota in RE patients showed distinct changes compared with healthy controls (Ahn et al., 2012). Several other studies also demonstrated that the oral microbiome may change in patients with gastric cancer, colorectal cancer pancreatic cancer and throat Cancer (Yang et al., 2009; Ahn et al., 2012; Farrell et al., 2012; Chen et al., 2015; Wang et al., 2019). Thus, it is logical to expect changes in the oral microbiota of RE patients.

Indeed, our study found changes in oral microbial taxa in RE patients as compared to health controls. At the phylum level, we found that *Proteobacteria*, *Bacteroidetes* and *Spirochaetes* were different from that of the controls in oral microbiome. Interestingly, a study reported alterations in *Bacteroidetes, Proteobacteria, Fusobacteria*, and *Spirochaetes* at the phylum level were correlated with Barrett’s esophagus and RE and concluded that inflammation may be associated with diversity microbiome in the distal esophagus (Yang et al., 2009). Although the specimen for two studies were different (oral cavity and esophagus), the altered microbes identified have been largely consistent.

At the genus level, our research found that the abundance of *Prevotella, Veillonella, Megasphaera, Peptostreptococcus, Atopobium, Oribacterium, Eubacterium*, and *Lachnoanaerobaculum* were higher, and *Neisseria, Streptococcus, Rothia, Granulicatella, Gemella, Aggregatibacter, Treponema, Campylobacter, Filifactor, Corynebacterium*, and *Lactivibrio* were lower in RE patients. In support to this, a recent study identified the alterations in *Veillonella, Prevotella, Neisseria*, and *Fusobacterium* in patients with RE in the distal esophagus than that of normal esophagus (Liu et al., 2013).

There also seems to be inconsistency between our study and previous reports. For instance, Blackett et al. (Blackett et al., 2013) and colleagues found that *Campylobacter* was significantly enriched in GERD and Barrett’s esophagus (BE) than in the controls and esophageal adenocarcinoma. However, our data suggested that *Campylobacter* in the oral saliva was significantly lower than that of controls at the genus level. It is not surprising, given the fact that the samples were from different sites and there are major differences in the diets of the populations studied.

## Conclusions

Our study found for the first time that the oral microbial composition in patients with RE show significant differences with that or health controls, and provide specific microbial features as potential biomarkers or the diagnosis of RE.

## Data Availability Statement

The data generated for this study can be found in NCBI using the accession number PRJNA639327.

## Ethics Statement

The studies involving human participants were reviewed and approved by the Medical Ethics Association of Luoyang Central Hospital Affiliated to Zhengzhou University. The patients/participants provided their written informed consent to participate in this study. Written informed consent was obtained from the individual(s) for the publication of any potentially identifiable images or data included in this article.

## Author Contributions

BW and YZ wrote this manuscript. HC and QZ designed the study. TY and YY tested and analyzed the data. YX provided the technical assistance. All authors contributed to the article and approved the submitted version.

## Funding 

This work was supported in part by the Henan Medical Science and Technology Tackling Plan, China (2018020892).

## Conflict of Interest

The authors declare that the research was conducted in the absence of any commercial or financial relationships that could be construed as a potential conflict of interest.
